# Hypogammaglobulinemia and infections in patients with multiple sclerosis treated with anti-CD20 treatments: a systematic review and meta-analysis of 19,139 multiple sclerosis patients

**DOI:** 10.3389/fneur.2024.1380654

**Published:** 2024-04-18

**Authors:** Anas Elgenidy, Nagham Nader Abdelhalim, Mohammed Al-mahdi Al-kurdi, Lobna A. Mohamed, Mohamed M. Ghoneim, Ahmed Wagdy Fathy, Hazem Khaled Hassaan, Ahmed Anan, Omar Alomari

**Affiliations:** ^1^Faculty of Medicine, Cairo University, Giza, Egypt; ^2^Karl-Jaspers-Klinik, Bad Zwischenahn, Germany; ^3^Faculty of Medicine, University of Aleppo, Aleppo, Syria; ^4^Faculty of Medicine, Menoufia University, Shibin Al Kawm, Egypt; ^5^Faculty of Medicine, Alexandria University, Alexandria, Egypt; ^6^Faculty of Medicine, Kafrelshikh University, Kafrelsheikh, Egypt; ^7^Faculty of Medicine, Benha University, Benha, Egypt; ^8^Faculty of Medicine, Suez Canal University, Ismailia, Egypt; ^9^Hamidiye International School of Medicine, University of Health Sciences, Istanbul, Türkiye

**Keywords:** anti-CD20, meta-analysis, multiple sclerosis, hypogammaglobulinemia, infections

## Abstract

**Background:**

Recent years have seen the emergence of disease-modifying therapies in multiple sclerosis (MS), such as anti-cluster of differentiation 20 (anti-CD20) monoclonal antibodies, aiming to modulate the immune response and effectively manage MS. However, the relationship between anti-CD20 treatments and immunoglobulin G (IgG) levels, particularly the development of hypogammaglobulinemia and subsequent infection risks, remains a subject of scientific interest and variability. We aimed to investigate the intricate connection between anti-CD20 MS treatments, changes in IgG levels, and the associated risk of hypogammaglobulinemia and subsequent infections.

**Method:**

PubMed, Scopus, Embase, Cochrane, and Web of Science databases have been searched for relevant studies. The “R” software utilized to analyze the occurrence of hypogammaglobulinemia, infections and mean differences in IgG levels pre- and post-treatment. The subgrouping analyses were done based on drug type and treatment duration. The assessment of heterogeneity utilized the *I*^2^ and chi-squared tests, applying the random effect model.

**Results:**

Thirty-nine articles fulfilled our inclusion criteria and were included in our review which included a total of 20,501 MS patients. The overall prevalence rate of hypogammaglobulinemia was found to be 11% (95% CI: 0.08 to 0.15). Subgroup analysis based on drug type revealed varying prevalence rates, with rituximab showing the highest at 18%. Subgroup analysis based on drug usage duration revealed that the highest proportion of hypogammaglobulinemia occurred in individuals taking the drugs for 1 year or less (19%). The prevalence of infections in MS patients with a focus on different infection types stratified by the MS drug used revealed that pulmonary infections were the most prevalent (9%) followed by urinary tract infections (6%), gastrointestinal infections (2%), and skin and mucous membrane infections (2%). Additionally, a significant decrease in mean IgG levels after treatment compared to before treatment, with a mean difference of 0.57 (95% CI: 0.22 to 0.93).

**Conclusion:**

This study provides a comprehensive analysis of the impact of anti-CD20 drugs on serum IgG levels in MS patients, exploring the prevalence of hypogammaglobulinemia, based on different drug types, treatment durations, and infection patterns. The identified rates and patterns offer a foundation for clinicians to consider in their risk-benefit.

**Systematic review registration:**

https://www.crd.york.ac.uk/prospero/display_record.php?RecordID=518239, CRD42024518239.

## 1 Introduction

Multiple sclerosis (MS) is a complex autoimmune disorder affecting the central nervous system, characterized by demyelination and nerve function impairment (1). MS predominantly affects young adults, typically beginning between 20 to 40 years of age, and is a significant contributor to neurological disability in this age group (2). The disease presents with diverse clinical manifestations and has a multifactorial etiology involving genetic and environmental factors. The main aspect of MS pathology involves the formation of demyelinating lesions, which are predominantly found in the white and grey matter of the brain, along with the spinal cord (1). The immune system and inflammation play crucial roles in the neurodegenerative process of MS (3). From a clinical perspective, MS can exhibit two primary trajectories: relapsing or progressive (4). The most frequent presentation involves relapsing-remitting MS (RRMS), characterized by discrete episodes of neurological dysfunction, followed by partial, complete, or no recovery. However, it is important to acknowledge the growing body of evidence suggesting the presence of progression independent of relapse activity (3, 4). With time, the frequency of RRMS relapses generally diminishes. However, a gradual decline often emerges, leading to a continuous worsening of symptoms, a phase known as secondary progressive MS (SPMS) (1). Diagnosis is based on clinical manifestations, radiological findings (notably MRI T2 lesions), and laboratory evidence (including cerebrospinal fluid-specific oligoclonal bands). These constituent elements collectively adhere to the guidelines outlined in the 2017 McDonald criteria (5).

In recent years, various therapeutic interventions, including disease-modifying therapies (DMTs), have been developed to manage MS effectively. DMTs, such as anti-cluster of differentiation 20 (anti-CD20) monoclonal antibodies, aim to modulate the immune response and ameliorate the disease course. These treatments have revolutionized MS management by targeting B cells, which play a pivotal role in the pathogenesis of MS (3).

The intriguing relationship between anti-CD20 treatments, including rituximab, ocrelizumab, ofatumumab, ublituximab, and immunoglobulin G (IgG) levels has garnered scientific interest. However, its noteworthy to mention that these treatments can lead to low levels of immunoglobulin, known as hypogammaglobulinemia (6). The impact on IgG levels and the subsequent risk of infections varies among individuals and types of MS. Different anti-CD20 therapies may also have variable impacts on IgG levels. While advancements in anti-CD20 treatments have greatly benefited patients, there are inconsistencies and heterogeneity in the available data regarding the connection between these treatments, IgG levels, and the risk of infections. Currently, there is a lack of standardized data to guide physicians in adjusting treatment dosages based on IgG levels. Addressing these knowledge gaps is crucial for enhancing the quality of life for individuals living with MS. However, results from studies in this area have differed.

This review aims to investigate the relationship between anti-CD20 MS treatments, IgG levels, and the risk of hypogammaglobulinemia and infections. By doing so, it aims to inform discussions on anti-CD20 MS treatment strategies and improve treatment decisions to prevent severe infections in patients.

## 2 Methods

The study was designed and reported with adherence to the Preferred Reporting Items for Systematic Reviews and Meta-analysis (PRISMA) guidelines (7). We submitted the research protocol for this systematic review to the International Prospective Registry of Systematic Reviews (PROSPERO) database^1^ and assigned the PROSPERO ID: CRD42024518239. All working group members agreed on the study protocol before beginning the literature search.

### 2.1 Search strategy and study selection

We conducted a comprehensive search across five electronic databases (Medline via PubMed, Scopus, Embase, Cochrane, and Web of Science) from their inception up to Jan 25, 2024, to identify relevant studies. We used the Medical Subject Headings (Mesh) database to retrieve the synonyms of our search strategy, and the terms were combined using “OR” and “AND” Boolean operators, following the Cochrane Handbook for Systematic Reviews (Chapter 4.4.4) (8). The search strategy utilized were as follows: (“Immunoglobulin OR globulin OR Antibody OR Ig OR AB AND (G OR GAMMA OR γ) OR Hypogammaglobulinemia OR Agammaglobulinemia”) AND ((Multiple OR disseminated OR “Acute Fulminating”) AND (sclerosis OR “encephalomyelitis disseminata” OR “ADEM”)) OR MS AND (“Rituximab OR Mabthera OR Anti-CD20 OR Rituxan OR Ocrelizumab OR Ublituximab OR Ofatumumab OR Riabni OR Ruxience OR Truxima OR GP2013 OR CD20 OR IDEC-C2B8 OR “IDEC C2B8”). The detailed search terms and results for each database are provided in Supplementary Table S1.

Following the removal of duplicates by the endnote software, two authors screened the retrieved studies independently based on our predefined eligibility criteria using titles and abstracts. Subsequently, the list of included studies was subjected to further scrutiny by two authors. Studies deemed relevant and any conflicts were subjected to full-text screening. Additionally, we conducted a manual search by reviewing the references of the articles included, and literature reviews for possible relevant studies.

### 2.2 Eligibility criteria

We included studies that met the following criteria: (1) all studies regardless of language or study type, and (2) studies that examined the effects of anti-CD20 monoclonal antibody treatments on serum IgG levels among diverse subtypes of MS patients. We excluded the following: (1) reviews, editorial correspondence, book chapters, animal and laboratory experiments, and *in vitro* inquiries, and (2) studies that did not explicitly mention serum IgG levels as a quantifiable factor. This meticulous approach ensures that the included studies meet high standards of quality and relevance.

### 2.3 Data extraction

Two authors independently extracted the data from the studies included entering the collected information into a pre-piloted Excel spreadsheet. To guarantee data accuracy and consistency, another author meticulously reviewed the completed extraction sheet, reconciled any discrepancies, and validated the precision of the data.

The data extraction encompassed several key elements, starting with study characteristics such as the first author’s name, publication year, study design, geographical location, and duration of the disease under investigation. Population characteristics, including sample size, gender distribution, age, duration of anti-CD20 treatment, type of MS, and interventions administered, were also recorded.

Furthermore, when applicable data on population characteristics were gathered based on after and before anti-CD20 administration, Expanded Disability Status Scale (EDSS) scores at the start and at the end of treatment, and different phases of the condition (PPMS, RRMS, SPMS). For the quantitative analysis, the same authors independently extracted information; the serum IgG levels in patients with MS, which was also extracted for different types of MS when applicable, and for different subtypes of anti-CD20 drugs data necessary to analyze the correlation of anti-CD20 treatment with serum IgG levels, and crucial variables for conducting the necessary statistical analysis. They also extracted data for different types of infections such as GIT, skin, pulmonary, and urinary. The data of interest were collected in the form of event, total, mean, and standard deviation. Data reported using different formats were converted into mean and standard deviation values using the website developed by McGrath et al. (9) which is an online tool used to facilitate the estimation of sample mean and standard deviation, ensuring consistency in the data presentation and analysis.

### 2.4 Methodology of the data analysis

Our meta-analysis was conducted using the meta-package of the R software (R version 4.1.0) (10), This package provided a systematic approach to extracting and analyzing data from the selected studies. We performed an initial analysis to calculate the percentage of occurrence of hypogammaglobulinemia among MS patients undergoing treatment with anti-CD20 drugs. Additionally, a subgroup analysis was performed based on drug type and treatment duration to identify specific effects. We also calculated the mean difference in IgG levels by comparing baseline (pre-treatment) and post-treatment levels among different drug types. Furthermore, we calculated the infections rate during treatment, including pulmonary, urinary tract, gastrointestinal, and skin/mucous membrane infections. The proportion of patients developing specific infections for each drug was calculated, providing insights into infection patterns associated with anti-CD20 therapies.

We assessed the heterogeneity using the *I*^2^ and chi-squared tests and applied the random effect model. Heterogeneity was considered substantial when *I*^2^ was more than 50% at a *p*-value <0.05. Mean differences were reported with 95% confidence intervals for continuous data. Publication bias was assessed visually using a funnel plot when enough studies were included in the analysis (*n* ≥ 10). For all the outcomes, we conducted leave-one-out meta-analyses, in which each of the meta-analyses was repeated by removing a single study, one at a time, to demonstrate how each study influences the total estimate.

### 2.5 The methodological quality of the included studies

Two authors independently assessed the quality of the included studies and resolved any disagreements through discussion with a third author. The updated Cochrane risk of bias tool for randomized trials (ROB 2.0) was used to evaluate the risk of bias in the RCT studies (11). The risk of bias table covered biases related to randomization, deviations from expected interventions, missing data, outcome measurement, and selection of reported results. Each trial was categorized as high risk, some concerns, or low risk based on the assessment. In addition, we used the NIH tool to assess the quality of cohort and cross-sectional studies, which consisted of 14 questions related to the research methodology (12). Each question is answered with yes, no, or unclear. The risk bias of the included case-control studies was determined using the Newcastle–Ottawa Scale (NOS), a star system consisting of nine questions, with a point awarded for each answer marked with an asterisk (13).

## 3 Results

### 3.1 Study selection

**Figure 1:**
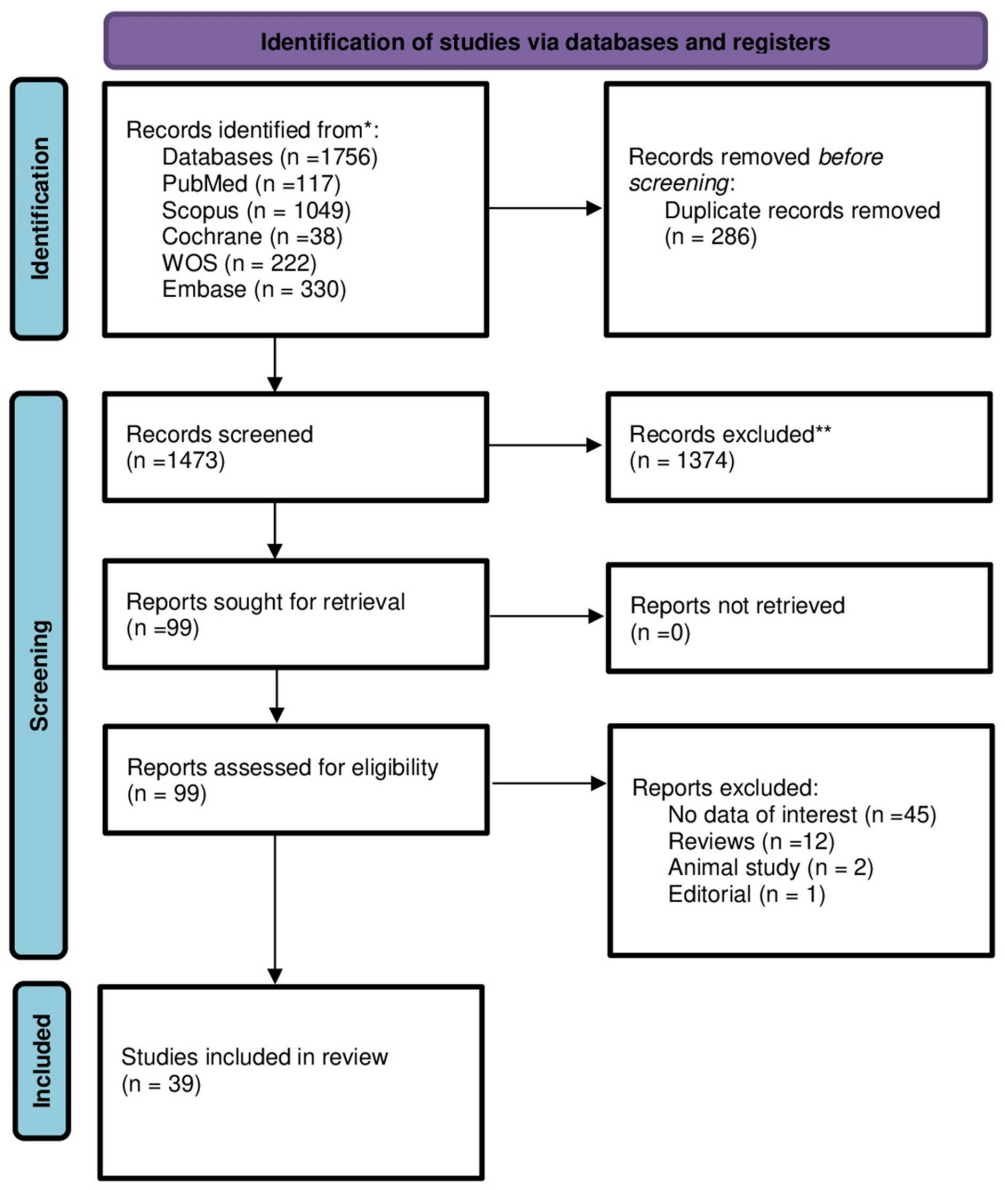
PRISMA flow diagram for new systematic reviews showing the selecting process of the articles which included searches of databases and registers only.

Following full-text screening, we identified 39 articles that met our inclusion criteria and were included in our review. These articles included six randomized clinical trials, 29 observational cohort, one was cross-sectional study, and three case control studies (Figure 1) (14–52).

### 3.2 Study characteristics

The relevant studies encompassed a total of 20,501 MS patients. Geographically. These studies were conducted in various countries, including France, Italy, Sweden, the USA, Qatar, Croatia, Switzerland, Norway, Denmark, Canada, Australia, Spain, Sweden, Greece, and the United Kingdom, providing a global perspective on MS treatments. The interventions investigated involve a range of anti-CD20 drugs, including therapies like rituximab, ocrelizumab, ofatumumab, and ublituximab. The patient populations studied in these diverse MS investigations represented a wide range of ages, typically spanning from the late 20s or early 30s to beyond 70 years. Sex distribution across the studies varies, with the percentage of female participants typically ranging from approximately 45 to 75%, indicating a higher representation of females across the studies. The studies also included different MS subtypes, such as Relapsing-Remitting MS (RRMS), Secondary Progressive MS (SPMS), Primary Progressive MS (PPMS), and Progressive-Relapsing MS (PRMS), ensuring a comprehensive representation of the disease spectrum. The duration of the disease, measured in years since onset or diagnosis, covers a broad spectrum, ranging from a few years to several decades. EDSS scores, indicating disability levels, vary across the studies, generally spanning from low scores indicative of mild disability to higher scores associated with more severe disability. In some studies, the specific type of immunosuppressive therapy used among the previously mentioned three drugs was not specified, so we referred to them collectively as “Anti CD20 drugs” in our analysis. A summary of the baseline characteristics of the included studies is presented in Table 1. To simplify the results of our subgroup analysis, we have provided a table summarizing the different correlations explored between the aforementioned factors (Table 2).

**Table 1:** Baseline characteristics of papers included in the meta-analysis.

Paper name and year	Study type	Country	Intervention drug	Dose	Mode of administration	Mean age	Sex Female (%)	No. MS patients	MS types					EDSS
									RRMS	SP MS	PP MS	PR MS	Disease duration	
**Full papers**														
Hawker 2009	Randomized double-blind placebo-controlled multicenter trial	US and Canada	Rituximab	1,000 mg every 24 weeks, through 96 weeks (4 courses)	Intravenous	50.1 (9.0)	81 (55.1)	292	—	—	—	—	Since onset = 9.0 (6.8) Since diagnosis = 3.8 (4.2)	**Baseline** Mean (SD) = 4.7 (1.4) **Change from baseline to week 96** Mean (SD) = 0.33 (1.0)
Kappos 2011	Phase 2, randomized, placebo-controlled, multicenter trial	58 patients = North America 120 patients = East-Central Europe and Asia 34 patients = Western Europe 8 patients = Latin America	Ocrelizumab 600	1st cycle = two 300 mg doses 15 days apart 2nd cycle = 600 mg	Intravenous	35.6 (8.5)	35 (64)	55	—	—	—	—	Years (range) since onset = 6.5 (0.5–20.5) Years (range) since diagnosis = 3.6 (0.1–16.5)	**Baseline** **Mean (SD) = 3.5 (1.5)**
			Ocrelizumab 2000	1st cycle = two 1,000 mg doses 15 days apart 2nd cycle = 1,000 mg	Intravenous	38.5 (8.7)	38 (69)	55	—	—	—	—	Years (range) since onset = 7.7 (0.25–28.0) Years (range) since diagnosis = 4.4 (0.1–19.2)	**Baseline** **Mean (SD) = 3.4 (1.3)**
			Interferon Beta-1a followed by ocrelizumab	1st dose interferon = 30 μg every week Following doses ocrelizumab = two 300 mg doses 15 days apart	Interferon = intramuscular Ocrelizumab = intravenous	38.1 (9.3)	32 (59)	54	—	—	—	—	Years (range) since onset = 5.3 (0.8–35.2) Years (range) since diagnosis = 3.3 (0.1–20.2)	**Baseline** **Mean (SD) = 3.1 (1.5)**
Graves 2014	Retrospective cohort	USA	Rituximab	375 mg/m^2^ once weekly/four doses (4.9% of patients) (72% of patients)1 g/two weeks apart (13% of patients) two doses 500 mg/two weeks apart	Intravenous	46.1 (13.6)	72 (63)	114	27	45	17	9	Mean (SD) = 15 (9.9)	**Baseline** Median = 6 Range = 2–8
Salzer et al. 2016	Retrospective cohort	Sweden	Rituximab	(Possible initial high loading dose of 1,000–2,000 mg divided into 2 infusions given within 1 month) 500 or 1,000 mg rituximab IV every 6–12 months	Intravenous	42.6 (11.1)	545 (66.3)	822	557	198	67	—	11.3 (8.5)	**Baseline** **Median (range) = 3 (0–9)**
Montalban et al. 2017	Randomized double-blind placebo-controlled multicenter trial	Multicenter	Ocrelizumab	600 mg	Intravenous	44.7 (7.9)	237 (48.6)	488	—	—	488	—	2.9 (3.2)	**Baseline** **Mean (SD)** = 4.7 (1.2)
Gracia-Canibano 2019	Retrospective observational cohort	Qatar	Ocrelizumab	1st dose = two 300 mg doses two weeks apart Following doses = 600 mg/6 months	Intravenous	35.4 (10.5)	28 (46.7)	60	57	3	—	—	Mean (SD) = 7.8 (6.8)	**Baseline** Mean = 2.3 (SD = 2.6) IQR = 4.0 **At 12 months follow up** Mean = 2.2 IQR = 2.4
Zoehner 2019	Retrospective cross-sectional study	Switzerland	Anti-CD20 (NS)	—	—	Median = 38 IQR = 29–53	150 (66.4)	226	175	28	23	—	—	**Baseline** Median = 2.8 IQR = 1.5–5
		Greece				Median = 35 IQR = 30–44	63 (62.4)	101	101	—	—	—	—	**Baseline** Median = 2.0 IQR = 1–2.3
Bauthman 2020	Retrospective longitudinal observational	France	Anti-CD20 (NS)	—		43.9 (9.4)	25 (54.3)	46	23	9	14	—	—	**At start of treatment mean** Primary progressive = 5.6 (1) Relapsing remitting = 2.3 (1.3) Secondary progressive = 5.9 (1) **At 12 months mean** Primary progressive = 5.9 (1.2) Relapsing remitting = 2.2 (1.5) Secondary progressive = 5.7 (1.6)
Evertsson 2020	Retrospective cohort	Sweden/USA	Rituximab	1st dose = 500 or 1,000 mg Following doses = 500 mg/5–7 months	Intravenous	44.0 (11.7)	222 (71.3)	311	225	86	—	—	Mean (SD) = 11.3 (8.87)	**Baseline** Median = 2.5 IQR = 2.125
			Ocrelizumab	1st dose = two 300 mg doses two weeks apart Following doses = 600 mg/5–7 months	Intravenous	49.8 (11.9)	95 (59.0)	161	161	—	—	—	Mean (SD) = 12.5 (8.32)	—
Vollmer 2020	Retrospective cohort	USA	Rituximab	First dose = 1,000 mg twice in two weeks Following doses = 1,000 mg/6 months	Intravenous	43 (12.5)	644 (67)	907	574	215	118	—	Mean (SD) = 9.1 (8.3)	—
Disanto 2021	Prospective observational cohort	Switzerland	Rituximab	500 mg/6 months	Intravenous	Median Age = 51.0 IQR = 37.5–57.0	44 (74.6)	59	37	22	—	—	Median = 10 IQR = 5–18	1 year prior to therapy Median = 4.0 At start of therapy Median = 4.0 After 12 months Median = 3.5
			Rituximab	1,000 mg/6 months	Intravenous									1 year prior to therapy Median = 4.0 At start of therapy Median = 3.5
Oksbjerg 2021	Retrospective cohort	Denmark	Anti-CD20 (ofatumumab, ocrelizumab and rituximab)	**Ofatumumab and ocrelizumab** First dose = 300 mg twice two weeks apart Following doses = 600 mg/6 months **Rituximab** First dose = 1,000 mg twice in two weeks Following doses = 1,000 mg/6 months	Intravenous	Median = 43 IQR = 34–51	266 (63.9)	416	343	36	18	—	Median years =1.2 IQR = 0.8–2.0	**At follow up** Median = 3 IQR = 2–4.5
			Anti-CD20 (ofatumumab, ocrelizumab and rituximab)/(discontinued)			Median = 42 IQR = 34–52	25 (80.6)	31	—	—	—	—	Median years = 12.7 IQR = 2.8–17.1	**At follow up** Median = 5 IQR = 2.5–6.5
Seery 2021	Retrospective cohort	Australia	Ocrelizumab	—	—	43.4 (10.6)	134 (72.4)	185	167	7	7	—	Mean (SD) = 10.65 (7.46)	**Baseline** Median = 2.0 IQR = 1–4
Torgauten 2021	Retrospective cohort	Norway	Rituximab	1st dose = 1,000 mg Following doses = 500 mg/6 months	Intravenous	42.3 (12.1)	255 (69.9)	365	320	23	22	—	**Since diagnosis** Mean (SD) = 5.3 (7.0)	**Baseline** Median (range) = 2 (0–8) **Change from baseline** Median (range) =0 (−3.0 to 2.5)
Habek 2022	Retrospective cohort	Croatia	Ocrelizumab	—		44.6 (9.5)	75 (67)	109	73	36	—	—	Mean (SD) = 8.5 (5.8)	**Baseline** Median = 3.5 Range = 0–7.0
Hauser 2022	Retrospective cohort	—	Ofatumumab	First dose = 20 mg at weeks 0, 1, 2 Follow up dose = 20 mg monthly starting week 4	Subcutaneous	38.7 (9.2)	1,345 (68.3)	1969	1869	100	—	—	Mean (SD) since onset = 9.0 (7.3) Mean (SD) since diagnosis = 6.4 (6.2)	**Baseline** Mean = 2.9 SD = 1.4
Perriguey 2022	Prospective observational study	France	Rituximab	First dose = 1,000 mg twice in two weeks Following doses = 1,000 mg/ 6 months	Intravenous	43.4 (12.9)	118 (62.8)	188	151	20	17	—	Median years = 10 Range = 0–36	**Baseline** Range = 0–8
Peters 2022	Retrospective, single-center observational study	USA	Rituximab/ocrelizumab	—		(Data is not exclusive to MS patients only)		261	—	—	—	—		
Capasso 2023	Prospective observational cohort	Italy	Ocrelizumab	1st dose = two 300 mg doses two weeks apart Following doses = 600 mg/6 months	Intravenous	47.8 (10.5)	38 (48.7)	78	38	9	31	—	Mean (SD) = 13.2 (8.6)	**Baseline** Median = 3.5 Range = 1.5–7.0 **Follow up 36.5 (6.8) months** median = 4.0 range = 1.5–8.0
Karlowicz 2023	Retrospective cohort	Norway	Rituximab	1st dose = 1,000 mg Following doses = 500 mg/6 months	Intravenous	42.1 (12.0)	398 (71.2)	556	515	—	41	—	Mean (SD) = 8.2 (9.1)	**Baseline** Mean (SD) = 2.2 (1.8)
Veronica Mears 2023	Retrospective Cohort	USA	All	—	—	48.3 (13.6)		184	94	43	17	—	—	—
			Rituximab			49.3 (14.3)	26 (81.25)	32	2	—	—	—	—	—
			Ocrelizumab			48.1 (13.5)	97 (63.81)	152	92	43	17	—	—	—
**Abstracts and posters**														
López Ruiz et al. 2021	Retrospective cohort	Spain	Ocrelizumab	—	Intravenous	39.5 (8.7)	34 (65.3)	52	52	—	—	—	Mean (range) = 11.1 (1–27.3)	**Baseline** **Median (range) = 3.5 (1.5–6.5)** **At follow up at 19 months (SD15.1)** **Median (range) = 2.5 (1.5–6.5)**
Chang 2019	Retrospective cohort	USA	Ocrelizumab			Range 31 to 77	47 (65.3)	72	—	—	—	—	—	—
Defruss 2019	Retrospective multivariate analysis	Europe, Canada, Australia, South America, USA	Ocrelizumab OPERA trial	—	—	38.1 (9.3)	949 (65.5)	1,448	—	—	—	—	<5 years = 658 (45.4) 5–10 years = 405 (28.0) >10 = 13 (0.9)	<3.0, *n* (%) = 810 (55.9) 3.0–6.0, *n* (%) = 625 (43.2) >6.0, *n* (%) = 13 (0.9)
			Ocrelizumab ORATORIO trial	—	—	45.8 (8.1)	320 (49.7)	644	—	—	—	—	<5 years = 190 (29.5) 5–10 years = 288 (44.7) >10 = 147 (22.8) Missing = 19 (3.0)	<3.0, *n* (%) = 12 (1.9) 3.0–6.0, *n* (%) = 529 (82.1) >6.0, *n* (%) = 102 (15.8) Missing, *n* (%) = 1 (0.2)
Hallberg 2019	Retrospective cohort	Sweden	Rituximab	First dose = 1,000 mg Follow up dose = 500 mg/6 months for 3 years	Intravenous	—	—	251	—	—	—	—	—	—
Tran 2019	Retrospective cohort	USA	Ocrelizumab	—	—	—	—	207	—	—	—	—		
Tsao 2019	Retrospective cohort	USA	B-cell depleting therapy	—	—	—	—	80	—	—	—	—		
			Rituximab	—	—	—	—	32	—	—	—	—		
			Ocrelizumab	—	—	—	—	37	—	—	—	—		
			Both Rituximab and Ocrelizumab	—	—	—	—	11	—	—	—	—		
Seze 2020	Randomized controlled trial	—	Ofatumumab	1st dose: 60 mg Following doses = 20 mg every 4 weeks, starting from week 4	Subcutaneous	—	—	944	—	—	—	—		**Baseline** Range = 0–5.5
Vollmer 2020	Retrospective cohort	USA	Ocrelizumab	—	—	44.2	71 (75.5)	94	76	16	2	—	Mean years = 10	**Baseline** Median (IQR) = 5.5 (3.5–6.0)
Vollmer 2020	Retrospective cohort	USA	Anti-CD20 (NS)			(Data is not exclusive to MS patients only)	527							
Wiendl 2020	Retrospective cohort	—	Ofatumumab	1st dose = 20 mg, days 1, 7, and 14 Follow up doses = 20 mg/4 weeks from week 4 onwards	Subcutaneous			964						
Illiopoulou 2021	Prospective observational cohort	Sweden	Rituximab	—	—	—	60 (100)	60	—	—	—	—		
Khatri 2021	Prospective observational cohort	USA	Ocrelizumab			—	—	223	—	—	—	—		
Mehta2021	Retrospective cohort	—	Rituximab	1,000 mg/6.10 months	Intravenous	—	—	34	—	—	—	—		
Vollmer 2021	Retrospective cohort	USA	Ocrelizumab	—	—	44.4	181 (73.9)	245	200	37	8	—	Mean years = 9.6	
Wiendl 2021	Retrospective cohort		Ofatumumab					1969						
Nobile 2022	Retrospective cohort	Canada	Ocrelizumab	1st dose = two 300 mg doses two weeks apart Following doses = 600 mg/6 months	Intravenous	—	—	266	232	34	—	—		
Rempe 2022	Retrospective cohort	USA	Ocrelizumab Standard dosing (SD) regimen	1st dose = two 300 mg doses two weeks apart Following doses = 600 mg/6 months	Intravenous	—	—	65	—	—	—	—		
			Ocrelizumab B-cell based extended interval dosing (EID) regimen	Repeat infusions are delayed until there is evidence for B-cell repopulation	Intravenous	—	—	52	—	—	—	—		
Xavier 2022	Retrospective cohort		Rituximab					48						

EDSS, Expanded Disability Status Scale; PRMS, primary progressive multiple sclerosis; PRMS, progressive relapsing multiple sclerosis; RRMS, relapsing-remitting multiple sclerosis; SPMS, secondary progressive multiple sclerosis.Baseline Mean (SD): levels of IgG before the treatment as a mean and standard deviations. Baseline Median (range): levels of IgG before the treatment as a median and IQR. Change from baseline to week 96: change in the IgG levels during the treatment. At follow up: change in the IgG levels during the follow up.

**Table 2:** Summary Correlations Explored in Subgroup Analysis.

Subgroups	No. of entries	RR (95% CI)	*I*^2^ (%)	*p*_(interaction)_ (between subgroups)
**Total incidence of hypogammaglobulinemia**				
*By drug type*				
Ocrelizumab	11	0.11 [0.07; 0.16]	93	<0.01
Rituximab	12	0.18 [0.11; 0.28]	95	
Ofatumumab	3	0.02 [0.01; 0.02]	14	
Anti-CD20 (NS^*^)	6	0.10 [0.06; 0.15]	91	
*By level of hypogammaglobulinemia*				
More than or equal to 7	3	0.10 [0.03; 0.30]	95	0.03
6–7	11	0.14 [0.09; 0.23]	95	
4–6	10	0.05 [0.03; 0.09]	96	
2–4	2	0.01 [0.00; 0.10]	92	
Less than or equal to 2	2	0.03 [0.00; 0.35]	92	
*By treatment duration*				
Less than or equal to 1 year	4	0.19 [0.08; 0.40]	91	0.06
About 2 years	6	0.12 [0.05; 0.26]	95	
About 3 years	5	0.07 [0.02; 0.17]	98	
About 4 years	4	0.13 [0.06; 0.26]	93	
5 years	1	0.07 [0.06; 0.08]	—	
8 years	1	0.05 [0.03; 0.08]	—	
**By infection type**				
*Pulmonary infections*				
Ocrelizumab	5	0.24 [0.08; 0.54]	95	<0.01
Rituximab	3	0.01 [0.00; 0.29]	99	
Ofatumumab	3	0.07 [0.02; 0.22]	96	
Anti-CD20 (NS^*^)	1	0.03 [0.01; 0.05]	—	
*Urinary tract infections*				
Ocrelizumab	5	0.10 [0.03; 0.25]	89	0.21
Rituximab	3	0.03 [0.02; 0.06]	66	
Ofatumumab	2	0.02 [0.00; 0.14]	98	
Anti-CD20 (NS^*^)	1	0.03 [0.02, 0.05]	—	
*Gastrointestinal infections*				
Ocrelizumab	3	0.01 [0.00; 0.05]	87	0.10
Rituximab	3	0.04 [0.02; 0.09]	55	
Ofatumumab	1	0.03 [0.01; 0.07]	—	
Anti-CD20 (NS^*^)	1	0.01 [0.00; 0.03]	—	
*Skin and mucous membrane infections*				
Ocrelizumab	3	0.06 [0.01; 0.22]	79	0.01
Rituximab	2	0.00 [0.00; 0.02]	0	

Subgroups	Number of entries	MD (95% CI)	*I*^2^ (%)	*p*_(interaction)_ (between subgroups)
**Pre and post drug intake IgG levels**				
*By drug*				
Ocrelizumab	6	1.01 [0.45; 1.57]	52	<0.01
Rituximab	3	0.14 [−0.33; 0.61]	26	
Ofatumumab	2	−0.06 [−0.25; 0.14]	0	
Anti-CD20 (NS^*^)	2	0.91 [−0.25; 0.14]	82	
*By treatment duration*				
Less than or equal to 1 year	8	0.44 [0.19; 0.69]	0	<0.01
About 2 years	1	1.03 [−0.36; 2.42]	—	
About 3 years	3	0.65 [−0.83; 2.13]	90	
About 4 years	1	1.45 [0.32; 2.57]	—	

NS^*^, not specified.

### 3.3 Subgrouping by drug type and IgG level in serum

**Figure 2:**
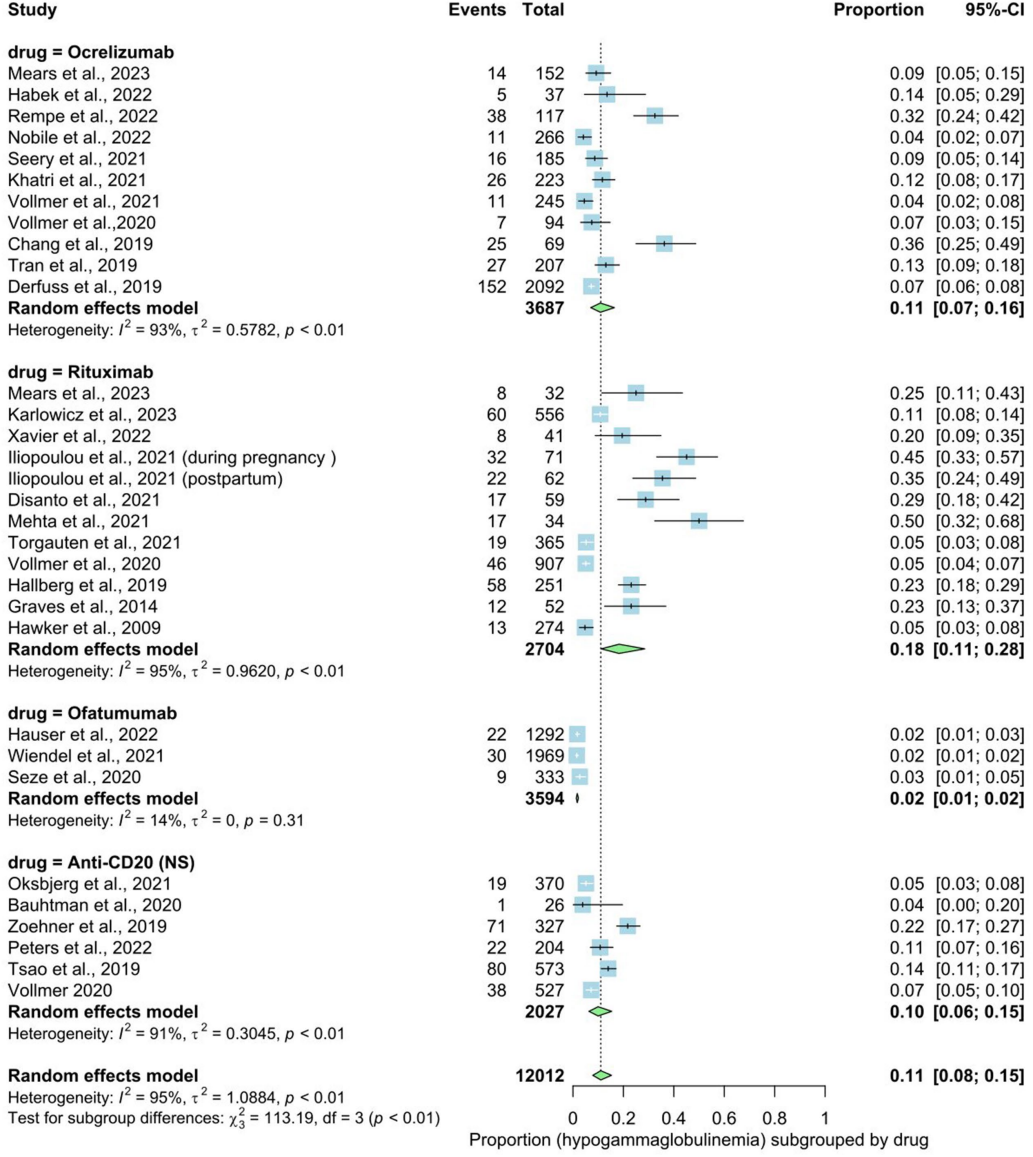
Analysis of drug type and IgG level in serum.

We identified 28 studies involving 12,012 patients that reported on the prevalence rate of hypogammaglobulinemia in patients of MS. The prevalence rate in the overall analysis was 11% (95% CI: 0.08 to 0.15), with substantial heterogeneity [*I*^2^ > 91% (*p* < 0.01)]. Examining subgroups based on drug type indicated that rituximab exhibited the highest prevalence at 18%, followed by ocrelizumab at 11%, non-specified anti-CD20 at 10%, and ofatumumab at 2%. The statistical test for subgroup differences yielded significance, indicating that the prevalence of hypogammaglobulinemia varies across drug types (rituxumab, ocrelizumab, ofatumumab, anti-CD20 (NS)) subgroups (*p* interaction <0.01) (Figure 2). The hypogammaglobulinemia thresholds determined by the included articles are listed in Table 3.

**Table 3:** Hypogammaglobulinemia thresholds determined by the included articles.

Subgrouping by reference point		
Study	Year	Reference point of hypogammaglobulinemia (g/L)
Salzer et al.	2016	6.2
Tran et al.	2019	6
Hallberg et al.	2019	5.65
Bauhtman et al.	2020	6
Seze et al. (16–19 years)	2020	5.49
Seze et al. (>19 years)	2020	7
Vollmer et al.	2020	7
Vollmer et al.	2020	5
Disanto et al.	2021	7.37
Wiendel et al.	2020	7
Oksbjerg et al.	2021	6.1
Iliopoulou et al.	2021	6.7
Wiendel et al.	2021	5.65
Khatri et al.	2021	6
Vollmer et al.	2021	5
Seery et al.	2021	5.52
Perriguey et al.	2022	7
Habek et al.	2022	7
Hauser et al.	2022	5.65
Peters et al.	2022	6
Xavier et al.	2022	6
Pukaj et al.	2022	7
Mears et al.	2023	6
Karlowicz et al.	2023	6
Capasso et al.	2023	7.37

### 3.4 Subgrouping by treatment duration

**Figure 3:**
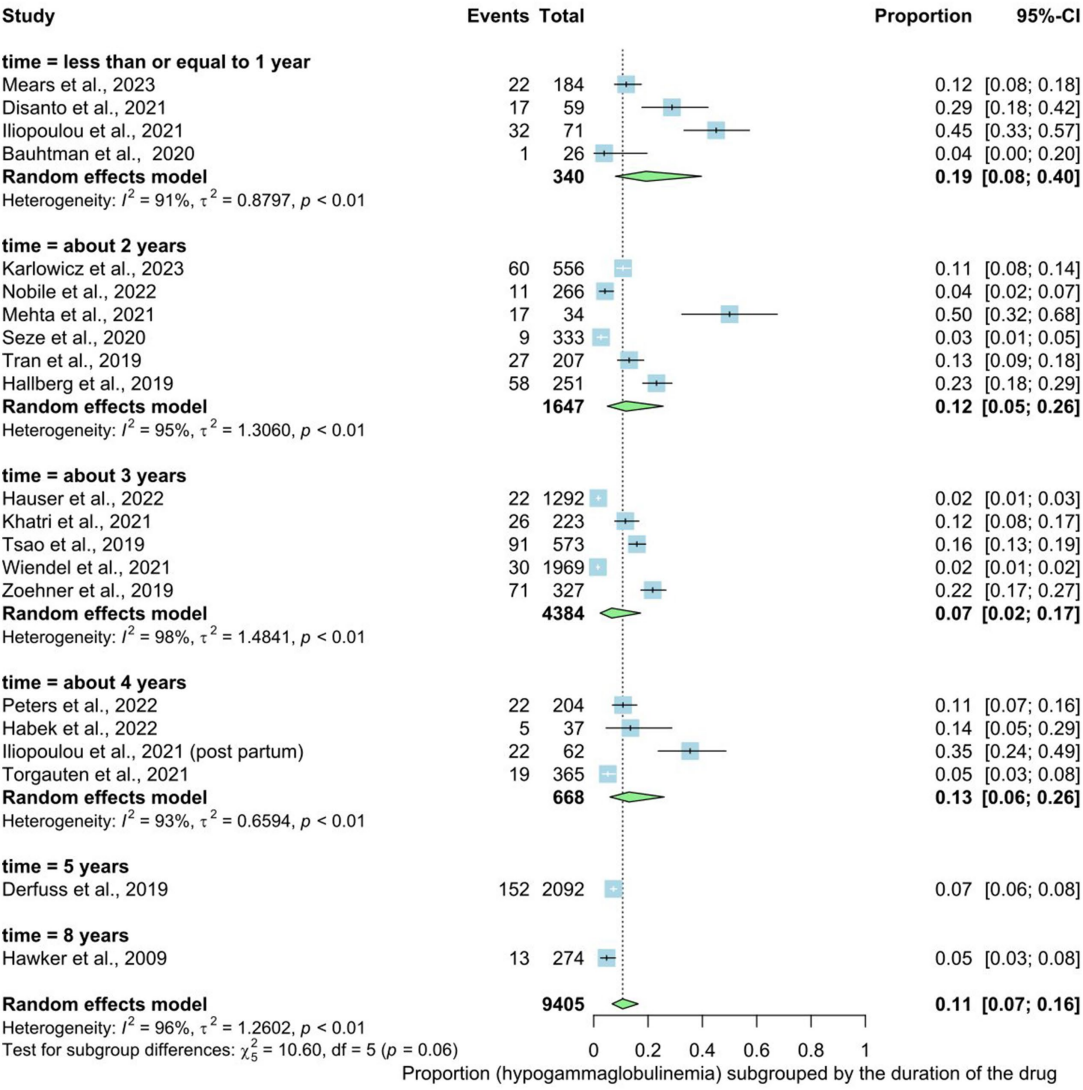
Analysis of IgG level and treatment duration.

We identified 20 studies involving 9,405 patients revealed that the duration of drug intake was a significant factor in hypogammaglobulinemia development. The prevalence rate of hypogammaglobulinemia in patients who take these drugs was 11% (95% CI: 0.07 to 0.16), with substantial heterogeneity [*I*^2^ > 96% (*p* < 0.01)] (Figure 3). Upon conducting subgroup analysis based on drug usage duration, it was observed that the highest proportion of hypogammaglobulinemia development occurred in individuals taking these drugs for 1 year or less (19%), followed by 4 years (13%), 2 years (12%), and 3 years (7%). There was no statistically significant difference between the six subgroups regarding duration of treatment with the specified disease modifying therapy (1 year or less, about 2 years, about 3 years, about 4 years, about 5 years or about 8 years) denoting that the proportion of hypogammaglobulinemia development does not vary majorly across different groups of drug intake duration (*p* interaction = 0.06).

### 3.5 Subgrouping by infection type

In this analysis, our aim was to identify prevalent infection types that occurred during the patients’ treatment. The analysis was stratified for each infection type based on the MS drug used during the study.

#### 3.5.1 Pulmonary infections

**Figure 4:**
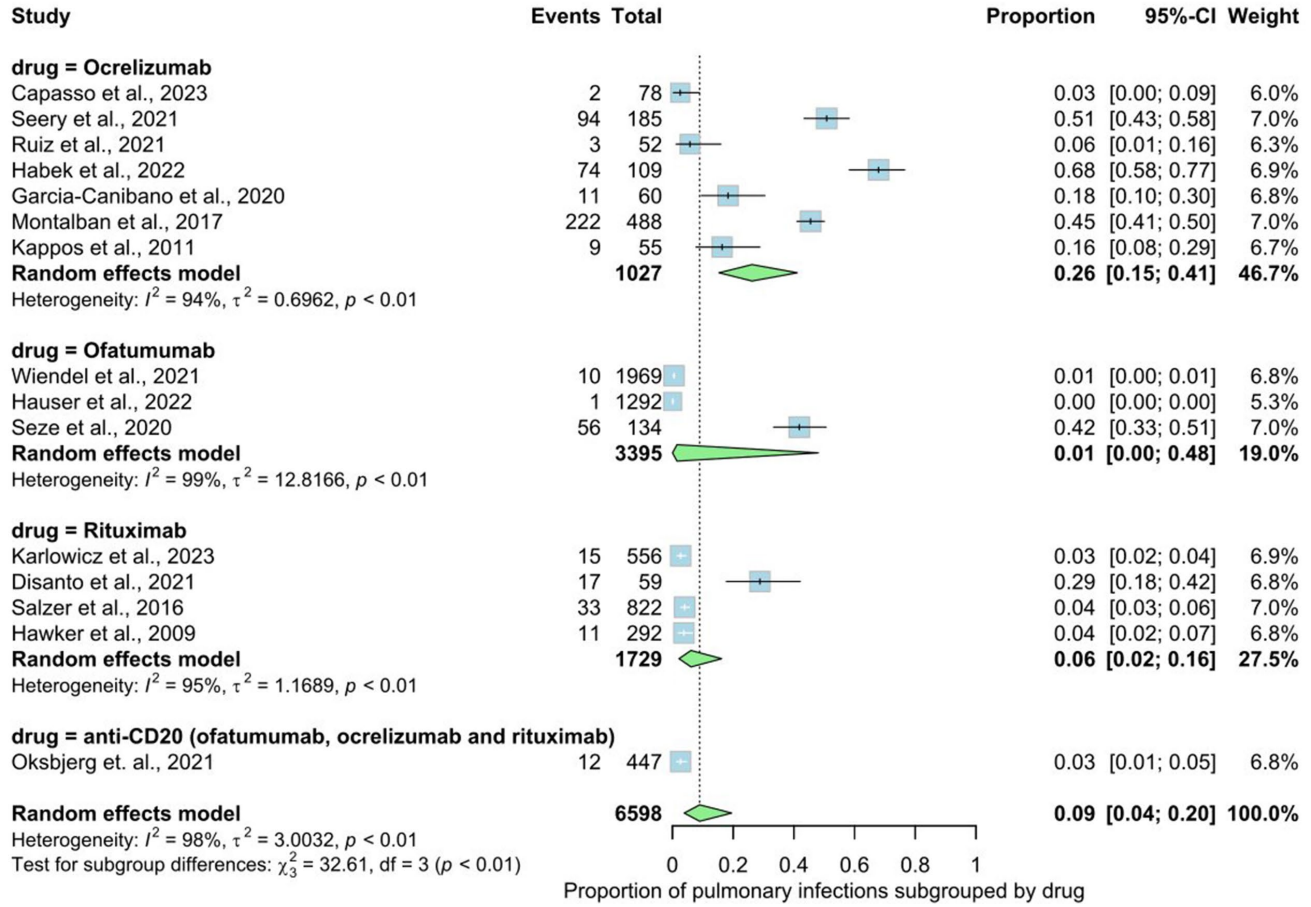
Analysis of Pulmonary infections among the included studies.

Fourteen studies involving 6,598 patients indicated the prevalence rate of pulmonary infections in MS patients. These infections ranged from upper respiratory tract issues like rhinitis and sinusitis to lower tract infections, including pneumonia requiring hospitalization. The prevalence rate in the overall analysis was 9% (95% CI: 0.04 to 0.20), with substantial heterogeneity [*I*^2^ > 98% (*p* < 0.01)] (Figure 4). Examining subgroups based on drug type indicated that patients who used ocrelizumab exhibited the highest prevalence of pulmonary infections at 26%, followed by rituxumab at 6%, non-specified anti-CD20 at 3%, and ofatumumab at 1%. The statistical test for subgroup differences yielded significance, indicating that the prevalence of pulmonary infections varies across drug types (rituxumab, ocrelizumab, ofatumumab), subgroups (*p* interaction <0.01).

#### 3.5.2 Urinary tract infections

**Figure 5:**
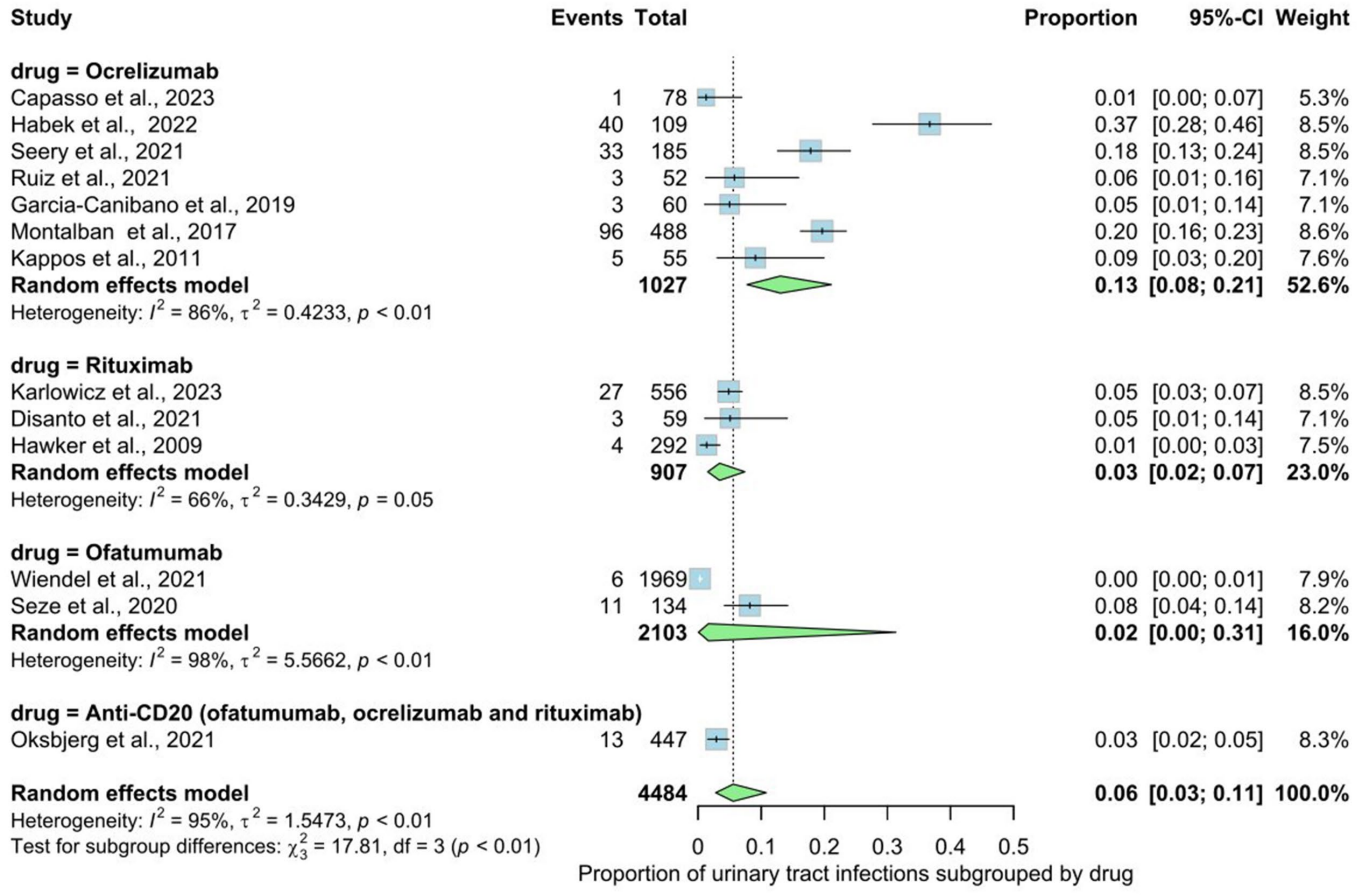
Analysis of Urinary tract infections (UTI) among the included studies.

Thirteen studies involving 4,484 patients indicated the prevalence rate of urinary tract infections (UTI) in MS patients. These infections ranged from upper to lower tract infections. The prevalence rate in the overall analysis was 6% (95% CI: 0.03 to 0.11), with substantial heterogeneity [*I*^2^ > 95% (*p* < 0.01)] (Figure 5). Examining subgroups based on drug type indicated that patients use ocrelizumab exhibited the highest prevalence of urinary infections at 13%, followed by rituxumab at 3%, non-specified anti-CD20 at 3%, and ofatumumab at 2%. The statistical test for subgroup differences did not yield significance, indicating that the prevalence of urinary infections does not vary across drug types (rituxumab, ocrelizumab, ofatumumab), subgroups (*p* interaction <0.01).

#### 3.5.3 Gastrointestinal infections

**Figure 6:**
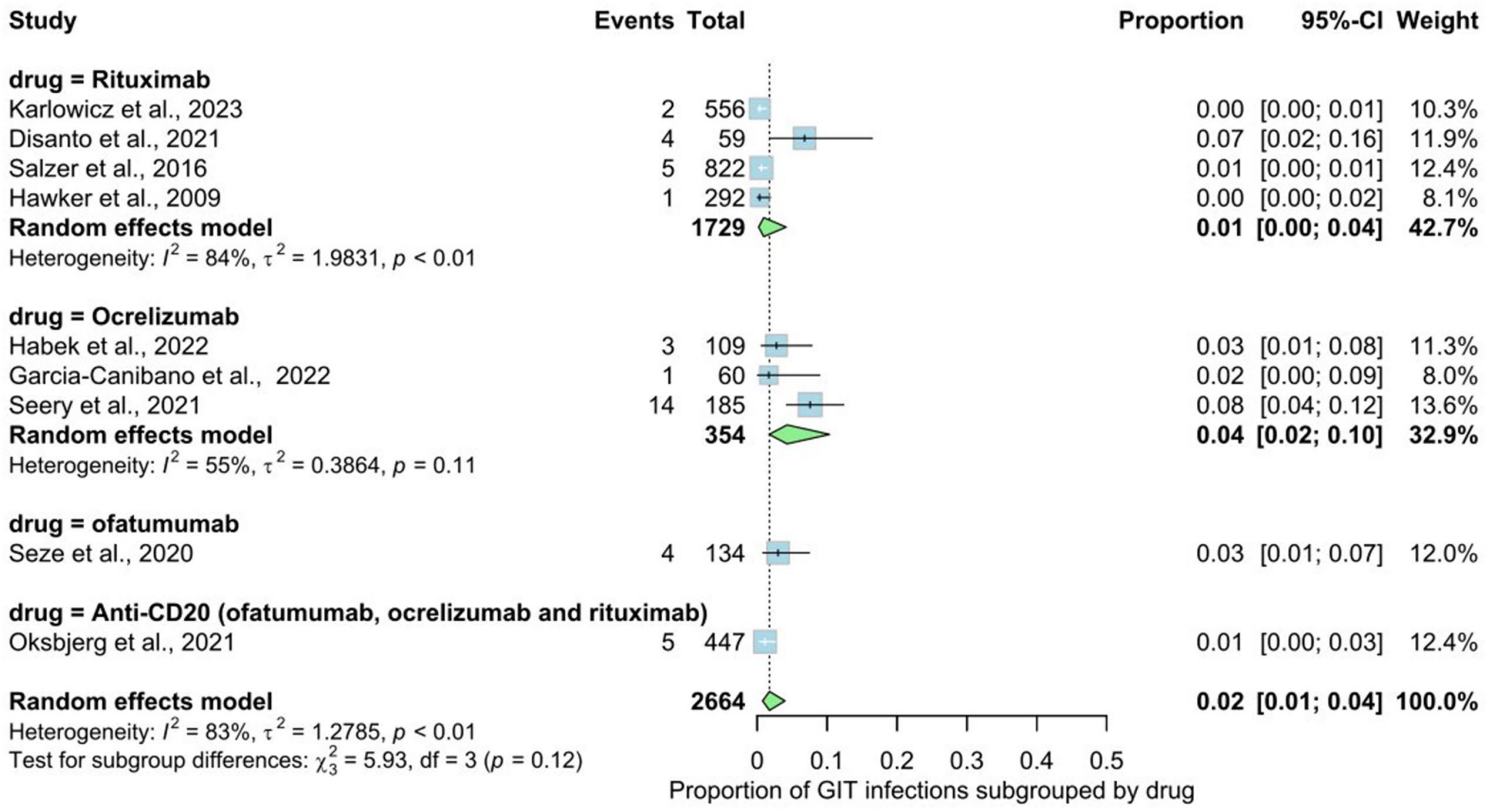
Analysis of Gastrointestinal (GIT) infections among the included studies.

Nine studies involving 2,664 patients indicated the prevalence rate of gastrointestinal (GIT) infections in MS patients. The prevalence rate in the overall analysis was 2% (95% CI: 0.01 to 0.04), with substantial heterogeneity [*I*^2^ > 79% (*p* < 0.01)] (Figure 6). Examining subgroups based on drug type indicated that patients use ocrelizumab exhibited the highest prevalence of gastrointestinal infections at 4%, followed by ofatumumab at 3%, rituxumab at 1%, and non-specified anti-CD20 at 1%. The statistical test for subgroup differences did not yield significance, indicating that the prevalence of gastrointestinal infections does not vary across drug types (rituxumab, ocrelizumab, ofatumumab), subgroups (*p* interaction = 0.12).

#### 3.5.4 Skin and mucous membrane infections

**Figure 7:**
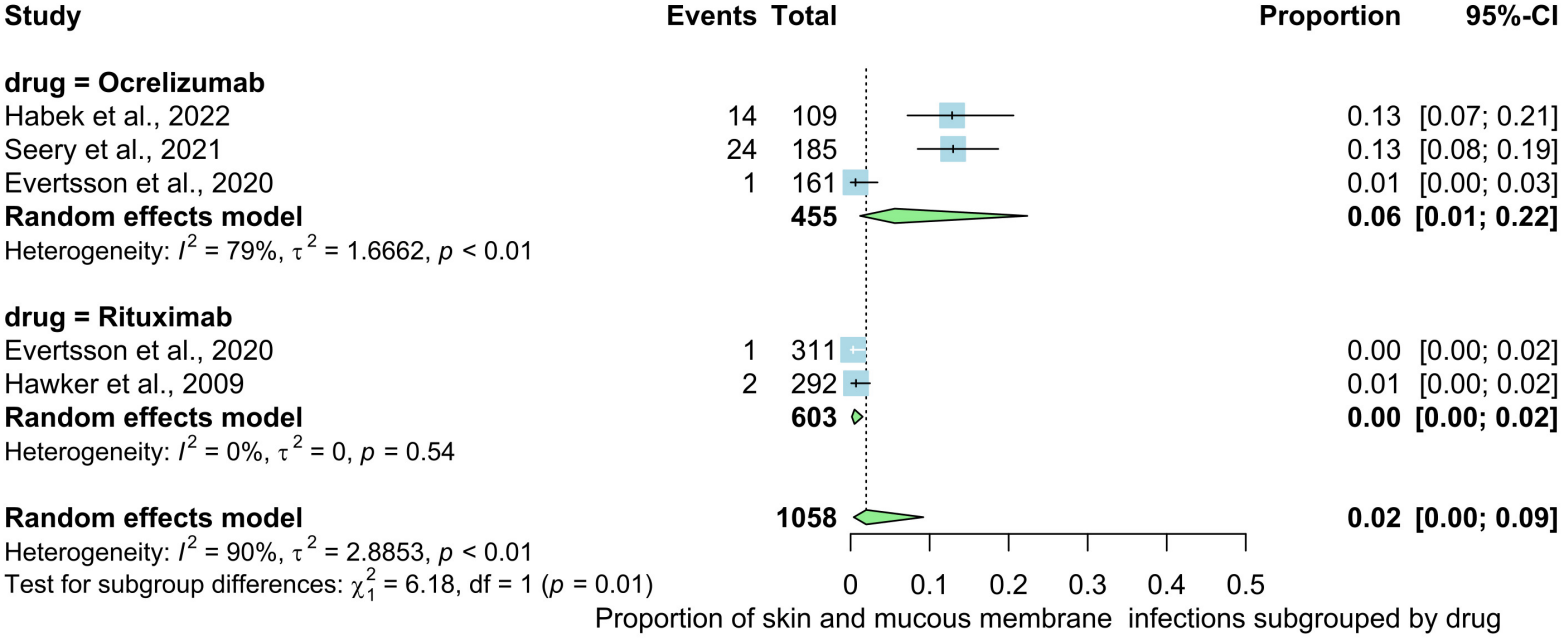
Analysis of Skin and mucous membrane infections among the included studies.

Five studies involving 1,058 patients indicated the prevalence rate of skin and mucous membrane infections in MS patients. The prevalence rate in the overall analysis was 2% (95% CI: 0.00 to 0.09), with substantial heterogeneity [*I*^2^ > 90% (*p* < 0.01)] (Figure 7). Examining subgroups based on drug type indicated that patients use ocrelizumab exhibited the highest prevalence of gastrointestinal infections at 6%, followed by rituxumab at 0%. The statistical test for subgroup differences yielded significance, indicating that the prevalence of skin and mucous membrane infections varies across drug types (rituxumab, ocrelizumab), subgroups (*p* interaction <0.01).

#### 3.5.5 Herpes virus infections

**Figure 8:**
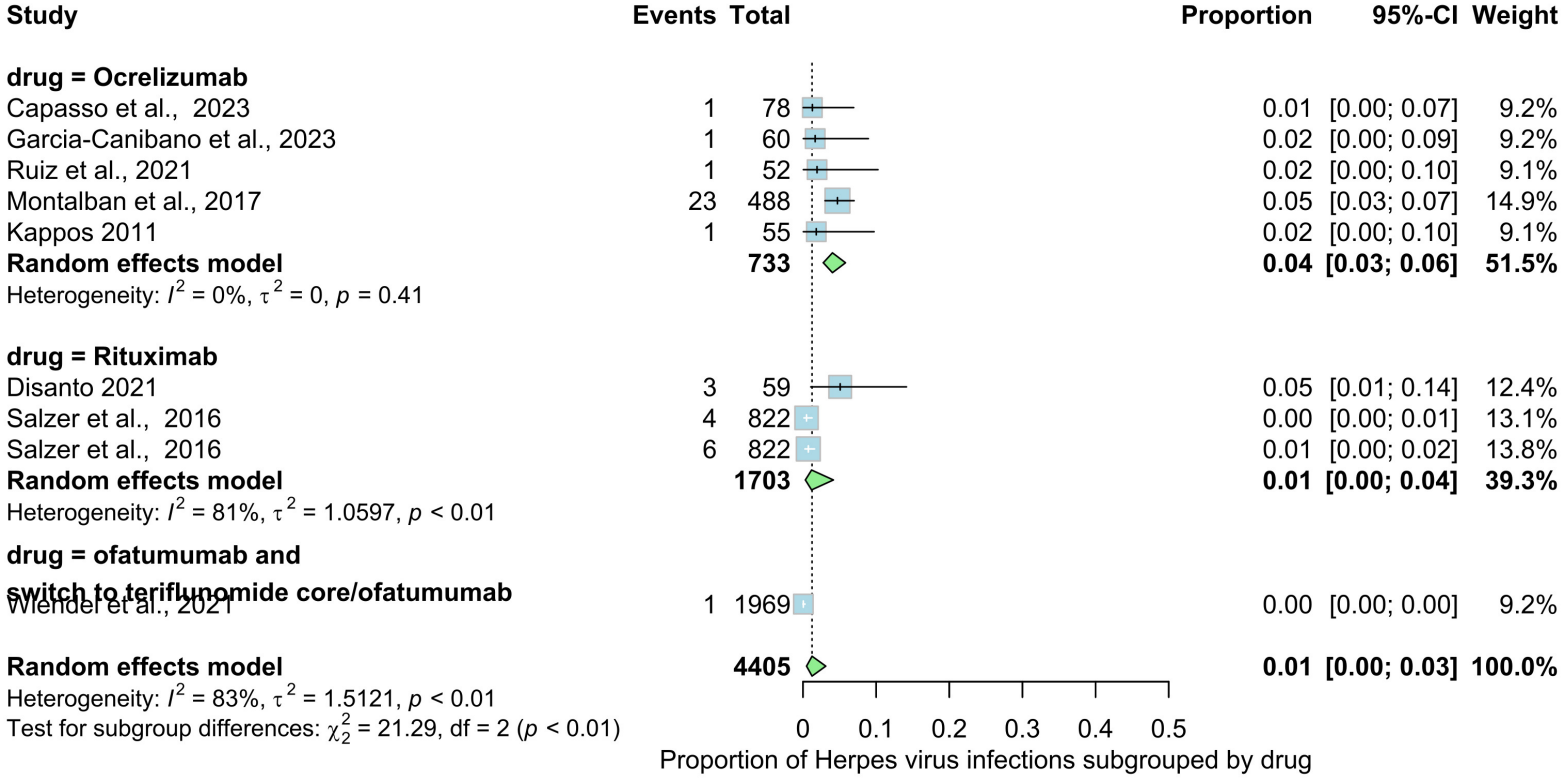
Analysis of Herpes Virus infection among included studies.

Nine studies involving 4,405 patients indicated the prevalence rate of herpes virus infections in MS patients. The prevalence rate in the overall analysis was 1% (95% CI: 0.00 to 0.03), with substantial heterogeneity [*I*^2^ > 90% (*p* < 0.01)] (Figure 8). Examining subgroups based on drug type indicated that patients use ocrelizumab exhibited the highest prevalence of gastrointestinal infections at 4%, followed by rituxumab at 1%; ofatumumab at 0%. The statistical test for subgroup differences yielded significance, indicating that the prevalence of skin and mucous membrane infections varies across drug types (rituxumab, ocrelizumab and ofatumumab), subgroups (*p* interaction <0.01).

### 3.6 Mean difference in IgG level pre-drug and post-drug intake

Our analysis aimed to investigate the relationship between the MS drugs and IgG levels drop by comparing the mean IgG before and after drug intake taking into account the drug type and duration of the drug intake.

**Figure 9:**
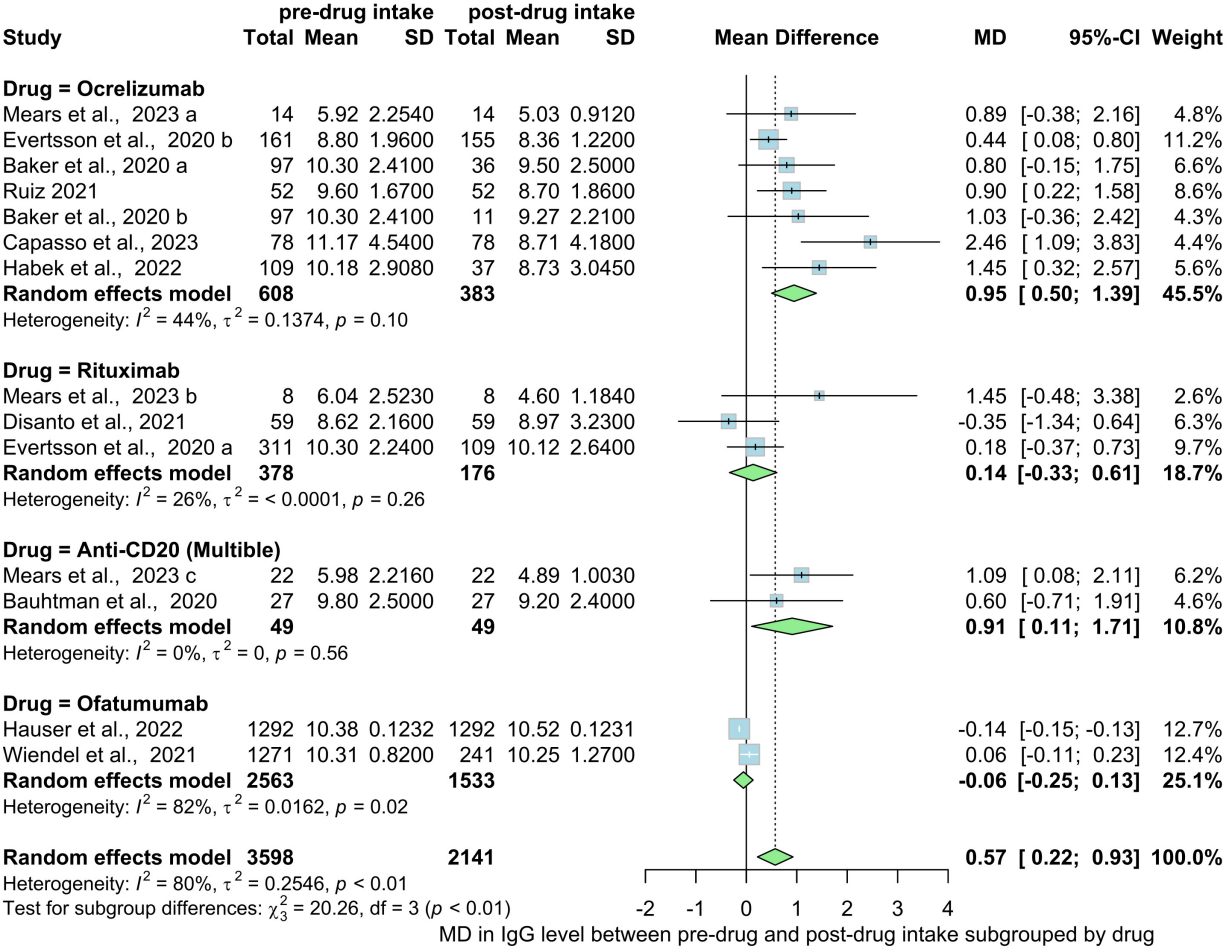
Mean difference in IgG level pre-drug and post-drug intake by drug type.

Ten studies involving 3,589 MS patients demonstrated significantly lower IgG level in post treatment group compared to pre-treatment. The mean difference was 0.57 (95% CI: 0.22 to 0.93), with substantial heterogeneity (*I*^2^ > 80%) (Figure 9). However, when subgrouping by different drugs only Ofatumumab showed statistically significant IgG levels drop of −0.06 mean difference (−0.25; 013, *I*^2^ = 82%, *p* = 0.02) while ocrelizumab, rituximab and anti-CD20 all showed no statistical significance (*p* = 0.10, 0.26, 0.56 respectively). Subgroups of different drugs [rituxumab, ocrelizumab, ofatumumab, anti-CD20 (NS)], Figure 9 shows statistical significance in mean differences of serum IgG levels decrease (*p* interaction = <0.01). The statistical test for subgroup differences yielded significance, indicating that the mean differences of serum IgG levels vary across drug types [rituxumab, ocrelizumab, ofatumumab, anti-CD20 (NS)], subgroups (*p* interaction <0.01).

**Figure 10:**
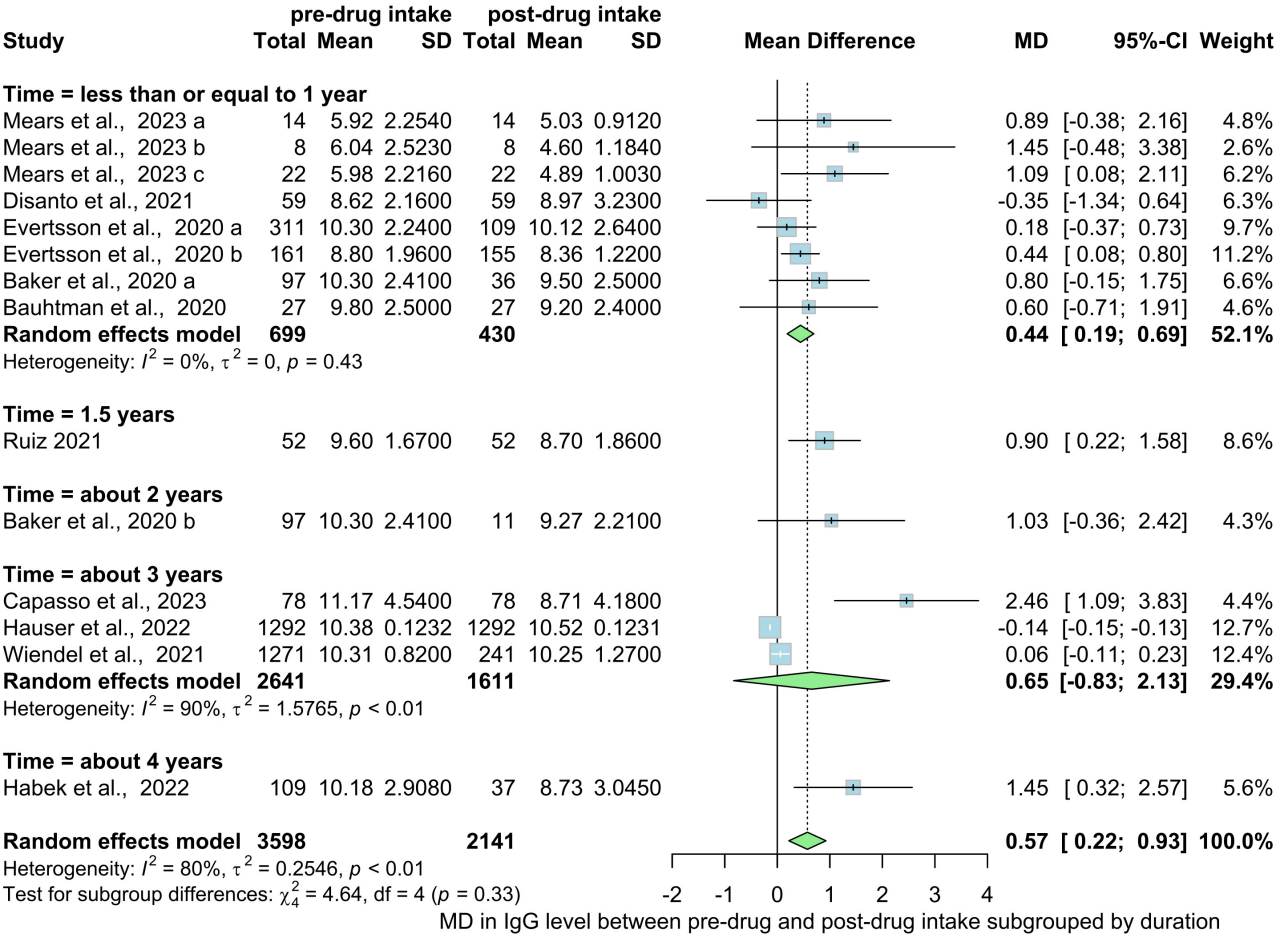
Mean difference in IgG level pre-drug and post-drug intake by time.

Regarding the duration of drug intake, the overall showed a mean difference of 0.57 in IgG level between pre-drug and post-drug intake (0.17; 0.93, *I*^2^ = 79%, *p* < 0.01) (Figure 10). Subgroup analysis based on drug usage duration revealed that drug intake for one year or less showed no significant change when comparing pre to post mean IgG level (*p* = 0.43). Subgroups of treatment duration of about 3 years also show no statistically significant mean difference of IgG level. There were not enough studies available for drug intake durations of 2 years, 1.5 years or 4 years to apply the random effects model on the data. Subgroups of different treatment durations (1 year or less, 1.5 years, about 2 years, about 3 years, about 4 years) Figure 10 showed no statistical significance in mean differences of serum IgG levels decrease (*p* interaction = 0.33).

### 3.7 Funnel plot analyses: publication bias

The assessment of potential publication bias through funnel plots yielded consistently revealed no evidence of publication bias or asymmetry. Overall, funnel plot assessments consistently revealed no evidence of publication bias or asymmetry. In the subgroup analysis based on drug type and IgG levels in serum, the linear regression Egger’s test yielded no detectable funnel plot asymmetry (*p* = 0.5908). Similarly, the investigation into treatment duration and its relationship with hypogammaglobulinemia showed no funnel plot asymmetry, as indicated by the Egger’s test result of *p* = 0.9035. Furthermore, in the analysis of mean differences in IgG levels pre-drug and post-drug intake, the Egger’s test for the overall results once again demonstrated no funnel plot asymmetry (*p* = 0.9035).

### 3.8 Leave-one-out results

We conducted leave-one-out analyses to evaluate the impact of individual studies on the overall findings within each subgroup analysis. Across all subgroup analyses based on drug type, IgG levels in serum, treatment duration, and infection type, no single study significantly influenced the results. This suggests the robustness and stability of the reported data.

### 3.9 Quality assessment of included studies

We utilized various tools to assess the quality of the 36 studies included in our analysis. We used the ROB2 tool for six RCTs; three of them were rated as good quality (24, 26, 51), and the other three studies were of low quality (23, 36, 46) due to the limited available data in the studies. We used the NIH tool for 30 Cohort and cross-sectional studies, 19 of them were of good quality (14, 15, 17–22, 25, 27, 28, 32, 35, 37, 40, 41, 47, 50, 52), 11 studies were of fair quality (16, 29, 30, 38, 39, 42–45, 48, 49). Three studies were assessed using the Newcastle-Ottawa Scale, with two scoring nine stars (31, 33), and one scoring eight stars (34). We also encountered challenges with incomplete data in some conference abstracts, leading to a fair quality rating for many of the assessments. Further details can be found in the Supplementary material containing the tables of quality assessment.

## 4 Discussion

To our knowledge this is the first large population-based study investigating the intricate connection between anti-CD20 MS treatments, changes in IgG levels, and the associated risk of hypogammaglobulinemia and subsequent infections. In our comprehensive analysis, we examined 36 studies aiming to elucidate the link between the immunosuppressive impacts of various anti-CD20 monoclonal antibody drugs used in MS and the occurrence of hypogammaglobulinemia, along with the resulting infections. The overall analysis indicates a significant prevalence of hypogammaglobulinemia (11%) among MS patients take anti-CD20 drugs. Examining subgroups based on drug type indicated that rituximab exhibited the highest prevalence at 18%, followed by ocrelizumab at 11%, non-specified anti-CD20 at 10%, and ofatumumab at 2%. Chronic usage of anti-CD20 therapies, such as rituximab and ocrelizumab, has been associated with a decline in immunoglobulin levels, particularly IgG and IgM. The decrease in IgG levels may persist even after the discontinuation of therapy. Also, anti-CD20 therapies, particularly ofatumumab and ocrelizumab, are effective in treating MS but pose a higher risk of infections.

Various disease-modifying therapies for MS differ in mechanisms, efficacy, and safety profiles, with a focus on targeting the immune response (53). Anti-CD20 therapies such as rituximab, ocrelizumab, ofatumumab, and ublituximab have proven effective and well-tolerated in MS patients through clinical trials, thereby expanding treatment options (54). Specifically, the anti-CD20 monoclonal antibodies ofatumumab and ocrelizumab, approved by the US Food and Drug Administration (FDA) and the European Medicines Agency (EMA), demonstrate efficacy in relapsing multiple sclerosis (RMS) by delaying disease progression, reducing relapses, and limiting new lesion formations on brain scans (55, 56). Even though B-cell depleting (anti-CD20) therapy is effective in treating MS patients (32, 57), it comes with the highest infection risk among MS disease-modifying therapies (58).

As a complication of anti-CD20 therapy, a common occurrence is a decline in immunoglobulin levels. In the phase 3 trials for rituximab and ocrelizumab, there was an observed increase in the percentage of patients with serum IgG and IgM levels below the normal range, however, ofatumumab and ublituximab resulted in a rise in the proportion of patients displaying IgM levels below the lower limit of normal (LLN), while not impacting IgG levels (32, 53–59).

Hypogammaglobulinemia can manifest during prolonged anti-CD20 therapy (60, 61). One hypothesis suggests that despite anti-CD20 treatment not directly affecting IgG and IgM-producing plasma cells, it delays the regeneration of B-cells (54). Reports indicate the time required for B-cell regeneration post-treatment: 24 weeks for ofatumumab, 72 weeks (ranging 27–175) for ocrelizumab, and 70 weeks (ranging 0.1–75) for ublituximab (56). Even after 48 weeks from rituximab discontinuation, B-cell levels were merely 30.7% of baseline values (57). Another explanation involves certain B-cell subsets experiencing impaired reconstitution; for instance, post-rituximab therapy, regenerated B cells mainly comprise naïve B cells with fewer differentiated memory B cells (40, 62). These regenerated naive B cells display reduced ability to become plasma cells, leading to decreased IgG and IgA production while still capable of producing IgM (61). This suggests that the impact of anti-CD20 therapies on immunoglobulin levels might involve complex interactions between different types of B cells, affecting humoral immunity (54, 63, 64).

Our overall analysis shows a statistically significant decrease in IgG levels from pre- to post-drug intake and a significant hypogammaglobulinemia development rate among anti-CD20 drugs users. Ofatumumab showed a significant decrease in IgG levels, while ocrelizumab, and rituximab, did not show statistical significance. Saidha et al. (65) conducted a systematic review of clinical trials and real-world evidence (RWE) studies to investigate alterations in immunoglobulin levels in individuals diagnosed with RMS who underwent treatment with either ocrelizumab or ofatumumab, as well as to understand the correlation between changes in Ig levels and the occurrence of infections. The obtained results emphasized that the most frequently documented outcome was the variation in IgG levels. Among the ocrelizumab trial groups, four trial populations observed a decline in IgG levels over 24 to 336 weeks follow-up period. Conversely, in the five ofatumumab trial groups monitored for 104 to 168 weeks, a temporary drop in IgG levels occurred at week 48, but no sustained decrease was noted thereafter. In both ocrelizumab and ofatumumab trials, IgM displayed a declining trend over time. Additionally, a reduction in mean IgA levels was observed in the ocrelizumab treatment group throughout the 336-week follow-up period.

Our analysis also revealed that the duration of drug intake significantly influenced hypogammaglobulinemia development. However, no statistically significant difference was observed in the subgroup analysis based on different treatment durations, indicating that reduced IgG levels within 1 year did not necessarily lead to increased hypogammaglobulinemia with prolonged treatment. In a recent retrospective analysis of a considerable group of individuals with MS who were administered rituximab or ocrelizumab, 3.7% exhibited a decline in their IgG levels, dropping below 5 g/L on average after an exposure period of approximately 29.7 months (44).

Several studies in the literature have sought to investigate the potential association between low IgG levels and demographic characteristics of patients. In a study by Mears et al. (40) involving 184 patients treated with rituximab and ocrelizumab, 22 patients experienced hypogammaglobulinemia. Those with hypogammaglobulinemia were more likely to be aged ≥50 years and exhibited lower initial IgG levels. In another prospective observational study encompassing all patients with MS after undergoing a median of 5 (1–6) cycles of rituximab (32). The research revealed that age was linked to an increased risk of reduced IgG levels below 6 g/L, while there was no significant association with sex or a history of immunosuppressive treatment.

Evidence from certain clinical trials and observational studies has suggested an relationship between Ig antibody levels and infection rates, as well as infection severity in patients with MS (17, 65). Having an improved understanding of such risks is particularly relevant within the context of B cell-depleting therapeutic strategies because one of the main functions of B cells is antibody production. In a broader sense, people with MS taking B cell-depleting therapies that may interfere with the generation and/or release of Ig antibodies in response to infectious exposures may accordingly have a greater risk for serious infections (65). Our study reports an overall infection rate of 11% among MS patients receiving anti-CD20 drugs with ocrelizumab shows the highest prevalence of pulmonary infections (24%), urinary tract infections (10%), gastrointestinal infections (4%), and skin and mucous membrane infections (6%). This infection rate represents the proportion of MS patients who developed hypogammaglobulinemia out of the total number of patients on anti-CD20 drugs.

For rituximab, multiple retrospective studies have indicated that within the MS patient cohort receiving rituximab, a subset displaying reduced levels of IgG experienced escalated rates of severe infections necessitating hospitalization, prolonged antibiotic therapies, or intravenous antibiotic interventions when compared to counterparts with higher IgG levels (44). Another three observational studies encompassing a range of 59 to 1,000 patients, undergoing up to 3.5 years of rituximab treatment, established an augmented infection risk associated with IgG deficiency in MS patients, whereas IgM deficiency did not exhibit a similar association (17, 32, 33). Notably, one study highlighted those patients manifesting diminished IgG levels encountered elevated rates of severe infections even before their IgG levels declined, attributed to factors such as advanced age, extended disease duration, and diminished CD19 count (expressed in pro-B cells, B cells, and short-lived plasma cells) (63). These findings emphasize the intricate interplay of various factors contributing to infection risk beyond solely IgG levels.

Screening for hypogammaglobulinemia is vital as it helps identify patients at risk of severe infections (66). Therefore, initiation of anti-CD20 therapy demands careful monitoring due to the increased potency of infections. However, the medical literature lacks a universally agreed upon threshold for defining hypogammaglobulinemia in MS studies (67). Various definitions of the lower limit of normal (LLN) for immunoglobulins (IgG and IgM) have been utilized across different clinical investigations. The LLN for these immunoglobulins can differ depending on factors such as patient age and the specific clinical laboratory used (67). The range of studies included in our analysis provides a diverse spectrum of reported values for defining hypogammaglobulinemia in MS. Across the studies, the reported cutoff values vary considerably, with the lowest being 5 mg/dL and the highest reaching 7 mg/dL. Considering this broad range, it is evident that there is substantial variability in the literature regarding the threshold for defining hypogammaglobulinemia. To suggest the most suitable cutoff value, a balance between inclusivity and clinical relevance must be struck. Based on the distribution of reported values, a cutoff between 6 and 7 mg/dL emerges as a pragmatic choice, capturing a significant portion of the reported data while maintaining clinical significance. Therefore, a cutoff value of approximately 6–7 mg/dL may offer a reasonable compromise in defining hypogammaglobulinemia in MS studies.

Finally, anti-CD20 therapies have shown a link to higher chances of severe COVID-19 infection and hospitalization, as indicated by real-world data. While there was speculation regarding hypogammaglobulinemia contributing to this heightened risk, a retrospective study involving 758 patients found no significant association between IgG levels below 700 mg/dL and COVID-19 (68). Additionally, in individuals with MS, factors such as advanced age, Black ethnicity, lack of ambulatory ability, existing health conditions, and the use of glucocorticoids have been linked to an increased likelihood of COVID-19 hospitalization or death (53).

This study has some limitations to be addressed. Notably, challenges arose from incomplete data within certain conference abstracts, prompting a fair quality rating for several assessments. Moreover, the study acknowledges the presence of moderate to high heterogeneity in some analyses, may be attributable to differences in populations, ethnicity, and study designs. Mitigation strategies, such as random-effects models and subgroup analyses, were employed, with leave-one-out analyses conducted to validate the precision of estimates across various subanalysis groups. The intricate nature of confounding factors, particularly those associated with infections, poses complexities to the analysis, acknowledging that the considerable challenge in drawing definitive conclusions due to the high degree of heterogeneity. Additionally, the apparent differing rate of hypogammaglobulinemia may be influenced by patient-specific factors that could have varied between the groups receiving different medications, such as disease severity, duration, and individual immune response variations. Further investigation into these potential confounders is warranted to better elucidate the relationship between anti-CD20 MS treatments, IgG levels, and the risk of hypogammaglobulinemia. Also, the study underscores a limitation concerning the scarcity of studies, notably for specific drugs like ofatumumab. The limited data for certain interventions necessitates caution in drawing definitive conclusions, and future research endeavors addressing these gaps could significantly enhance the depth of understanding in this domain.

## 5 Conclusion

In conclusion, the study reveals a noteworthy prevalence rate of hypogammaglobulinemia development among MS patients take anti-CD20 therapies, with rituximab demonstrating the highest prevalence of low IgG concentration when compared to other MS drugs. This study contributes valuable insights into the immunosuppressive effects, infection risks, and implications of anti-CD20 therapies in MS treatment. The identified relationships and patterns offer a foundation for clinicians to consider in their risk-benefit assessments and underscore the importance of ongoing monitoring and research to optimize therapeutic strategies and patient outcomes in the context of MS treatment.

## Data availability statement

The original contributions presented in the study are included in the article/Supplementary material, further inquiries can be directed to the corresponding author.

## Author contributions

AE: Writing – review & editing, Writing – original draft, Visualization, Validation, Software, Resources, Project administration, Methodology, Investigation, Funding acquisition, Formal analysis, Data curation, Conceptualization. NA: Writing – review & editing, Writing – original draft, Methodology, Investigation, Data curation. MA-k: Writing – original draft, Visualization, Resources. LM: Writing – original draft, Resources, Methodology, Investigation. MG: Writing – original draft, Resources, Project administration, Methodology. AF: Writing – original draft, Investigation. HH: Writing – original draft, Resources, Project administration, Investigation. AA: Writing – original draft, Resources, Methodology, Investigation. OA: Writing – review & editing, Writing – original draft, Visualization, Validation, Supervision, Software, Resources, Project administration, Methodology, Investigation, Funding acquisition, Formal analysis, Data curation, Conceptualization.

## Footnotes

^1^ www.crd.york.ac.uk/prospero/
