# Supplementary material for: Hypogammaglobulinemia and infections in patients with multiple sclerosis treated with anti-CD20 treatments: a systematic review and meta-analysis of 19,139 multiple sclerosis patients
Source: Front Neurol. 2024 Apr 18;15:1380654. doi: 10.3389/fneur.2024.1380654 (PMC11063306; doi:10.3389/fneur.2024.1380654)
Supplement: Supplementary file 1 [file Table_1.docx]

Supplementary Table 1: Search Strategy

| **Database** | **Results** | **Term** |
| --- | --- | --- |
| PubMed | **117** | #1((Immunoglobulin OR globulin OR Antibody OR Ig OR AB) AND (G OR GAMMA OR γ)) OR Hypogammaglobulinemia OR Agammaglobulinemia  2# ((Multiple OR disseminated OR “Acute Fulminating”) AND (sclerosis OR “encephalomyelitis disseminata” OR “ADEM”)) OR MS  3# (Rituximab OR Mabthera OR Anti-CD20 OR Rituxan OR Ocrelizumab OR Ublituximab OR Ofatumumab OR Riabni OR Ruxience OR Truxima OR GP2013 OR CD20 OR IDEC-C2B8 OR "IDEC C2B8") |
| Cochrane | 38 |  |
| Scopus | 1049 |  |
| WOS | 222 |  |
| Embase | 330 |  |
| **Total** | **1756** |  |
